# Fluorescence Quenching of Graphene Quantum Dots from Orange Peel for Methyl Orange Detection

**DOI:** 10.3390/nano15050376

**Published:** 2025-02-28

**Authors:** Weitao Li, Yang Liu, Xinglong Pang, Yuanhao Huang, Zeyun Dong, Qian Niu, Yuping Xiong, Shang Li, Shuai Li, Lei Wang, Huazhang Guo, Saisai Cui, Shenpeng Hu, Yuenan Li, Tiantian Cha, Liang Wang

**Affiliations:** 1 Textile and Garment Industry of Research Institute, Zhongyuan University of Technology, Zhengzhou 450007, China; liweitao@zut.edu.cn (W.L.); 2023117779@zut.edu.cn (Y.L.); 2023017750@zut.edu.cn (Y.H.); 2023117759@zut.edu.cn (Z.D.); 2022117681@zut.edu.cn (Q.N.); 2022117679@zut.edu.cn (Y.X.); lishang@zut.edu.cn (S.L.); 2024117032@zut.edu.cn (S.L.); 2023117756@zut.edu.cn (S.C.); 2023117775@zut.edu.cn (S.H.); 2023117761@zut.edu.cn (Y.L.); 2023017754@zut.edu.cn (T.C.); 2 Zhengzhou Key Laboratory of Smart Fabrics & Flexible Electronics Technology, Zhongyuan University of Technology, Zhengzhou 451191, China; 3 Institute of Nanochemistry and Nanobiology, School of Environmental and Chemical Engineering, Shanghai University, Shanghai 200444, China; pangxinglong@tsinghua-zj.edu.cn (X.P.); wangl@shu.edu.cn (L.W.); 4 Department of Environment, Yangtze Delta Region Institute of Tsinghua University, Jiaxing 314006, China

**Keywords:** graphene quantum dots, orange peel, fluorescence quenching, methyl orange dye detection, dye detection

## Abstract

Methyl orange (MO) is an organic synthetic dye widely used in laboratory and industrial applications. In laboratory settings, it serves as an acid–base indicator due to its distinct color change in both acidic and alkaline environments. Industrially, it is primarily utilized in the textile industry for its ultraviolet (UV) absorption properties. However, the discharge and leakage of methyl orange into the environment can cause severe ecological damage and pose potential carcinogenic and teratogenic risks to human health. Therefore, detecting and quantifying the concentration of methyl orange in various matrices is crucial. This study reports the synthesis of graphene quantum dots (GQDs) from orange peel as a precursor, using ethanol and dimethylformamide (DMF) as solvents. Cyan (c-GQDs) and yellow (y-GQDs) graphene quantum dots were synthesized through a bottom-up hydrothermal method. The difference in color is attributed to the redshift caused by the varying ratio of pyridine nitrogen to pyrrole nitrogen. These GQDs exhibited notable optical properties, with c-GQDs emitting cyan fluorescence and y-GQDs emitting yellow fluorescence under UV light. To investigate fluorescence quenching effects, nine commonly used dyes were tested, and all were found to quench the fluorescence of y-GQDs, with methyl orange having the most significant effect. The fluorescence quenching of orange peel-derived GQDs in the presence of methyl orange is attributed to poor dispersion in DMF solution. Additionally, the GQDs possess high specific surface area, abundant surface functional groups, and excellent electronic conductivity, which contribute to their effective fluorescence quenching performance. The average thickness of y-GQDs (the vertical dimension from the substrate upwards) was 3.51 nm, confirming their graphene-like structure. They emitted yellow fluorescence within the wavelength range of 450–530 nm. Notably, a significant linear correlation was found between the concentration of methyl orange and the fluorescence intensity of y-GQDs (regression coefficient = 0.9954), indicating the potential of GQDs as effective sensing materials for organic pollutant detection.

## 1. Introduction

The significance of water in sustaining life is unequivocal [1], as it constitutes a substantial component of all biomass. With the rapid development of industries such as papermaking and textiles, the pollution resulting from dye wastewater has become an increasingly pressing issue. Dye wastewater is characterized by high color intensity, large volumes, and resistance to degradation, making it one of the most challenging types of industrial effluent to treat in China. These wastewater streams possess complex compositions, which not only reduce water transparency but also lead to oxygen depletion, hinder the natural self-purification processes of water bodies, and negatively impact the growth of aquatic organisms [2,3]. When discharged into urban sewage systems and other environments, these dyes can degrade into toxic aromatic amines. These compounds are notoriously difficult to degrade, exhibit persistence in the environment, and pose significant threats to human health. They are known to irritate the eyes and skin and may even exhibit carcinogenic and teratogenic properties. Methyl orange, an organic synthetic dye commonly used in both laboratory and industrial applications, serves as an acid–base indicator in laboratory settings due to its color-changing properties in acidic and alkaline conditions. In industry, it is primarily employed in the textile sector, owing to its ability to absorb ultraviolet light. As a frequently encountered azo dye, methyl orange carries inherent risks of carcinogenicity and teratogenicity [4,5]. The direct discharge of methyl orange-contaminated wastewater thus poses substantial health risks to humans and other organisms. Consequently, the monitoring of methyl orange concentrations in aquatic environments is of paramount importance. Graphene quantum dots (GQDs) represent a novel class of zero-dimensional fluorescent carbon nanomaterials [6,7,8,9,10,11,12,13,14]. Due to their remarkable optical stability and tunability [15,16], graphene quantum dots (GQDs) demonstrate remarkable multifunctionality across a spectrum of applications, with electrocatalysis emerging as a pivotal area of research focus [17,18,19].

The in situ synthesis of near-atomic-layer nanostructures was achieved through a GQD-assisted hydrothermal method, demonstrating the promising potential for electrocatalytic applications [20]. A substantial body of literature has reported various synthesis methods for GQDs [21], which can generally be categorized into two main approaches based on the carbon source: top-down and bottom-up methods [22,23]. Hydrogen energy, as a pre-eminent contender in the realm of clean energy alternatives, exhibits substantial potential and constitutes a pivotal domain in the application spectrum of graphene quantum dots (GQDs) [24]. In the context of environmental sustainability, the utilization of urban waste materials has attracted increasing attention in recent years. In 2017, Professor Liang Wang’s research group at Shanghai University reported a cost-effective solvothermal method for synthesizing orange-emitting GQDs, and in 2018, they proposed the preparation of GQDs from coffee grounds. This innovative approach not only demonstrates environmental benefits but also marks a significant advancement in the field. Inspired by this pioneering work, the present study explores the feasibility and methods for synthesizing GQDs from orange peel waste. In contrast, the bottom-up method involves the use of small molecular carbon precursors, which self-assemble into GQDs through a series of chemical reactions. This method provides better control over the optical properties of the resulting quantum dots, enabling fine-tuning through parameters such as catalyst type, concentration, and reaction conditions. Due to their excellent photoluminescent properties, GQDs have been widely applied in fields such as ion and dye detection [25,26,27], serving as effective fluorescent probes for the selective detection of dye ions [28]. Furthermore, GQDs possess good water solubility and low toxicity and do not alter water quality during metal ion detection processes [29,30,31,32]. These characteristics make them not only safe for human use but also highly suitable for synthesizing fluorescent probes with strong optical stability [33].

**Scheme 1:**
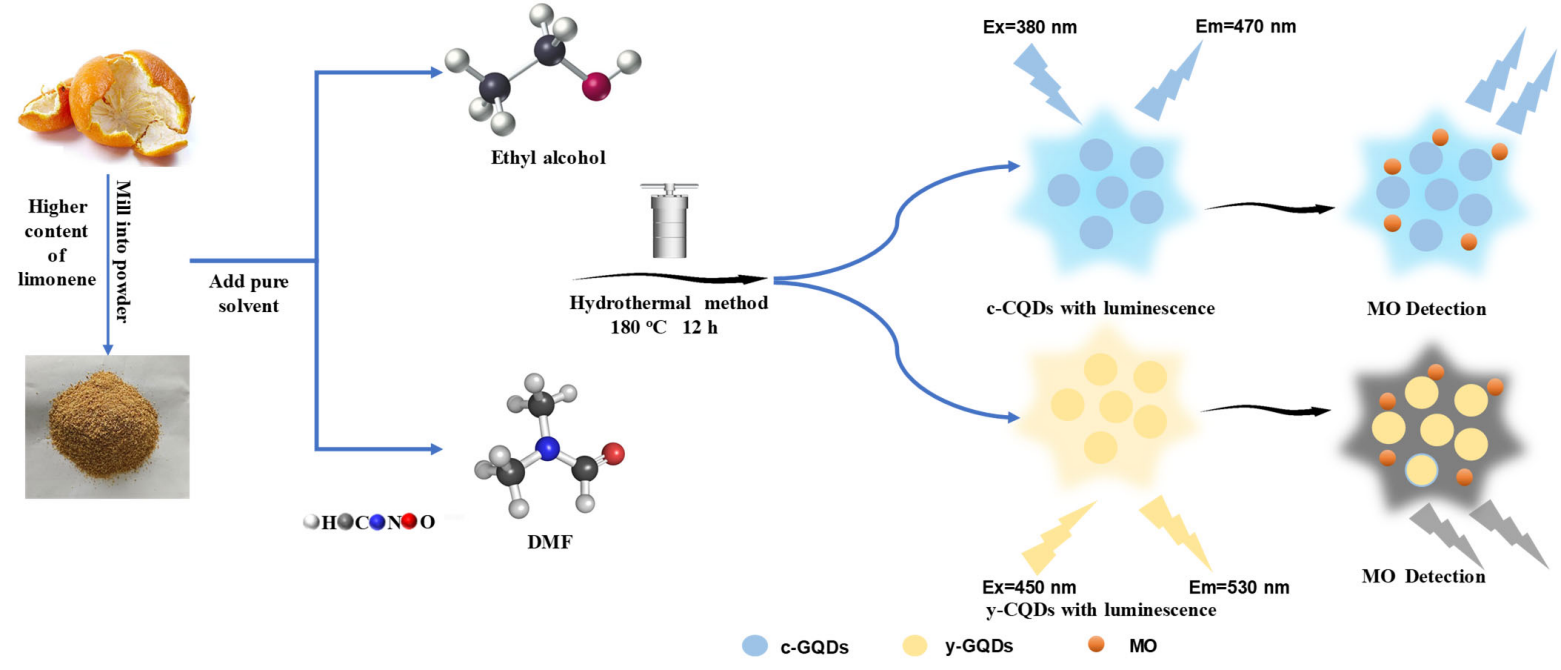
The synthesis and utilization of b-GQDs and c-GQDs for the detection of dyes.

In this study, the synthesis of GQDs using orange peel as a precursor material was investigated [34,35,36,37,38]. Unlike other precursors, orange peel is abundant in daily life, rich in limonene, and cost-effective [16,39], making it an ideal candidate for the preparation of carbon-based nanomaterials. Unlike most traditional solvothermal methods, this study employed DMF and ethanol as pure solvents for organic thermal reactions, simplifying the conventional solvothermal process by avoiding complex temperature and pressure control steps. During subsequent heating, cleavage and oxidation reactions occurred, forming carbon-based materials, which then underwent crosslinking and aggregation in the solvent, ultimately resulting in GQDs. Using DMF as a pure solvent facilitated the straightforward synthesis of yellow-emitting GQDs without the need for intricate process controls. In this work, a bottom-up hydrothermal method was employed to synthesize c-GQDs and y-GQDs using ethanol and DMF as pure solvents. The synthesized quantum dots exhibited excellent optical properties: c-GQDs emitted cyan fluorescence under UV light, while y-GQDs emitted yellow fluorescence. Upon mixing the quantum dot solution with a dye solution, significant quenching of y-GQDs fluorescence was observed with methyl orange, attributed to charge transfer, resonance energy transfer, or other molecular interactions between GQDs and methyl orange. These mechanisms collectively caused a notable reduction in or quenching of y-GQDs fluorescence. Furthermore, the separation of photogenerated electron–hole pairs in y-GQDs facilitated rapid electron transfer and enhanced electron–hole recombination, leading to fluorescence quenching. The experimental procedure is outlined in Scheme 1. Dried orange peels were manually ground in a mortar and then filtered to obtain the powder. A certain amount of the powder was placed in a reaction vessel, and ethanol and DMF were added. The mixture was reacted in an oven at 180 °C for 12 h and then cooled and filtered to obtain the desired quantum dots for further analysis.

## 2. Experiment and Facilities

In this experiment, all the chemical reagents and dyes were purchased from the China National Pharmaceutical Group (Sinopharm), Beijing, China, with a purity grade of AR (analytical reagent) and were used without the need for further purification. The deionized water used in the experiment was generated by the YL-100B-D water purification system, Shenzhen, China. Finally, the orange peel used in the experiment was sourced from commonly available fresh oranges.

### 2.1. Experimental Facilities

The height map of GQDs was measured by atomic force microscopy (FMP-3D Infinity, Oxford, UK), the characteristic peak of GQDs was measured by confocal Raman spectrometer (Horiba Xplora Plus, Shanghai, China), and the infrared spectrum of GQDs was measured by a Fourier transform infrared spectrometer (Bruker TENSOR37, Shanghai, China). A transmission electron microscope (JEM-2010F, Japan Electronics Co., Ltd., Tokyo, Japan) obtained high-resolution TEM images, an X-ray diffractometer (Philips X ‘Pert Pro, Shanghai, China) obtained XPS atomic measurement spectrograms, fluorescence spectra were measured with a fluorescence spectrometer (RF-6000, Shimadzu, Shanghai, China), and UV spectra were measured with a UV–visible near-infrared spectrometer (Cary5000, Agilent, Beijing, China). Zeta potential was tested by a laser particle size analyzer (Ni comp 380 Z3000 SOP, Entegris, Billerica, MA, USA).

### 2.2. Preparation of GQDs

The fresh orange peel was dried and subsequently crushed (the results remained consistent under different drying times—for example, 2–4 h. Precisely 0.05 g of the resulting orange peel powder was weighed using an analytical balance and transferred into a 10 mL PTFE-lined container. Then, 10 mL of ethanol or DMF was added to this as the solvent, and the mixture was then placed into a reaction vessel. The vessel was positioned in an oven, where the temperature was maintained at 180 °C for 12 h to facilitate the reaction. Upon cooling to ambient temperature, the reaction mixture was removed. The resulting solution was then subjected to filtration through a microporous membrane with a 220 nm cutoff, yielding the GQDs solution. The final products were designated as c-GQDs (using ethanol as the solvent) and y-GQDs (using DMF as the solvent).

### 2.3. Dye Detection

In this study, a series of commonly used dye solutions—specifically Methyl Orange, Methyl Blue, Methylene Blue, Malachite Green, Rhodamine B, Rhodamine 6G, Azure Blue, Astrazon Brilliant Red 4G, and Quinine Sulfate—were selected to explore their influence on the fluorescence intensity of c-GQDs and y-GQDs. The concentrations of the dye solutions were standardized at 0.1 mM. A defined volume of each dye solution was then mixed with the c-GQDs and y-GQDs, and the resulting fluorescence quenching of the quantum dot dispersions was subsequently measured after the addition of the different dye solutions.

## 3. Results and Discussion

### 3.1. Topography Characterization of GQDs

**Figure 1:**
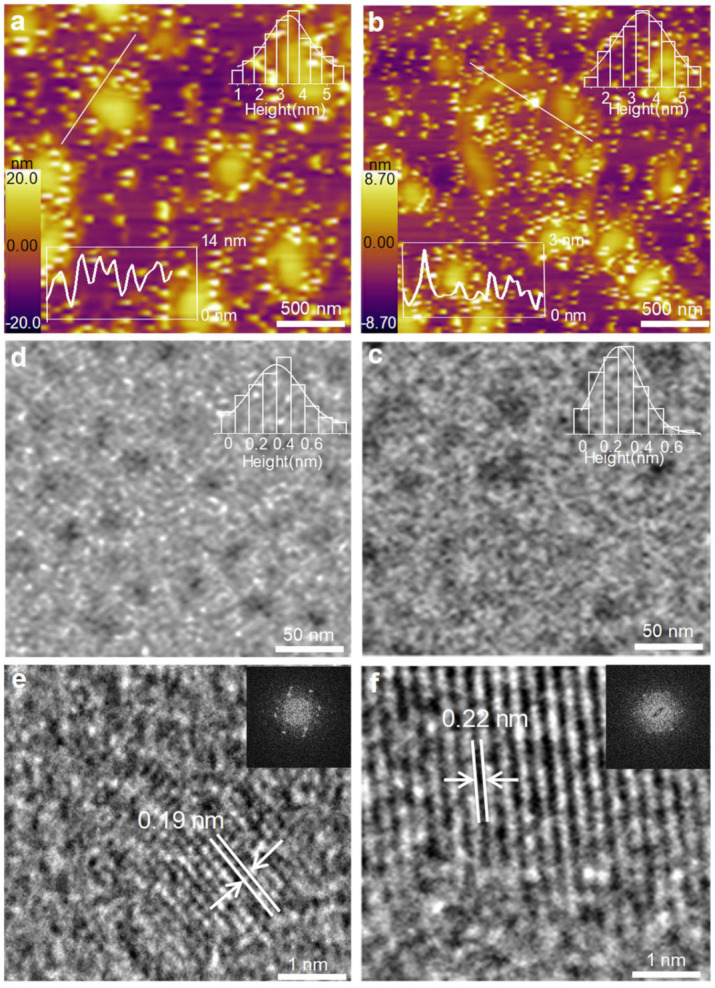
(**a**,**b**) AFM plots and height distribution of c-GQDs and y-GQDs. (**c**,**d**) TEM images and relative size distributions of c-GQDs and y-GQDs. (**e**,**f**) High-resolution TEM images of c-GQDs and y-GQDs.

Figure 1 illustrates the morphological characteristics of c-GQDs and y-GQDs, as observed through atomic force microscopy (AFM) and transmission electron microscopy (TEM). During the analysis, hundreds of individual samples were examined, and both the AFM height distribution and TEM size distribution were generated. Figure 1a,b present the AFM images and thickness distributions of c-GQDs and y-GQDs. The average thicknesses of c-GQDs and y-GQDs are 3.49 nm and 3.51 nm, respectively, indicating that both are composed of multilayer graphene sheets. Figure 1c,d display the TEM images and corresponding size distributions of c-GQDs and y-GQDs. The average particle size of c-GQDs is 0.25 nm, while that of y-GQDs is 0.41 nm. Both kinds of quantum dots exhibit a uniform size distribution, with y-GQDs showing a larger particle size compared to c-GQDs. This observation suggests that dimethylformamide (DMF) is more conducive to GQD growth than ethanol. The high-resolution TEM images of c-GQDs and y-GQDs are shown in Figure 1e,f, which also include their corresponding Fourier transform (FT) images. The high-resolution TEM clearly reveals the crystalline nature of both c-GQDs and y-GQDs, with a well-defined single-crystal structure. The lattice spacings of the two quantum dots are measured to be 0.19 nm and 0.22 nm, respectively. The FT images exhibit regular hexagonal symmetry, reminiscent of the benzene ring lattice structure typical of graphene, further confirming the high-quality single-crystal structures of both c-GQDs and y-GQDs. Notably, y-GQDs exhibit superior lattice symmetry, suggesting a higher degree of crystallinity compared to c-GQDs, which may account for their enhanced optical properties.

### 3.2. Structural Characterization of GQDs

**Figure 2:**
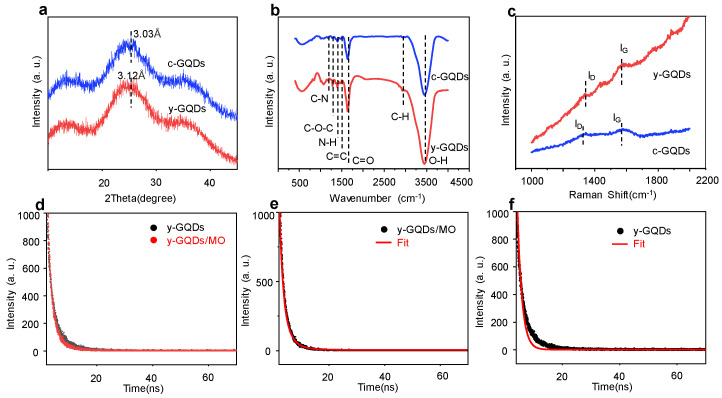
(**a**) XRD patterns of y-GQDs and c-GQDs, (**b**) FT-IR patterns of y-GQDs and c-GQDs, (**c**) Raman patterns of y-GQDs and c-GQDs. (**d**) Fluorescence lifetime of y-GQDs before and after MO addition. Exponential fitting of fluorescence lifetime of y-GQDs with MO (**e**) and without MO (**f**).

Figure 2a presents the X-ray diffraction (XRD) patterns of c-GQDs and y-GQDs, where a prominent diffraction peak is observed at approximately 26°, corresponding to interlayer spacings of 3.03 Å and 3.12 Å, respectively. The larger interlayer spacing in y-GQDs can be attributed to the use of the polar organic solvent DMF. DMF is a well-known polar solvent that can effectively dissolve a wide range of organic compounds, particularly those rich in oxygen-containing functional groups, such as cellulose, pectin, and polyphenols found in orange peel. The polar nature of DMF facilitates the dissolution of these components, thereby enhancing the synthesis efficiency of graphene quantum dots (GQDs). Naturally occurring organic compounds in orange peel, such as polyphenols, can spontaneously form graphene-based nanostructures under specific conditions. The presence of DMF ensures a more uniform distribution and interaction of these organic molecules in the solvent, promoting the self-assembly and nucleation processes of GQDs, ultimately resulting in high-quality GQDs. DMF was used as the nitrogen source to induce N-doping [40], which could also result in a redshift of the y-GQDs. This suggests that GQDs synthesized using DMF as a solvent exhibit superior crystallinity. Figure 2b displays the Fourier transform infrared (FT-IR) spectra of c-GQDs and y-GQDs, which were used to investigate the surface functional groups of the GQDs. The strong stretching vibration peaks at 3371 cm^−1^, 2962 cm^−1^, 1750 cm^−1^, 1593 cm^−1^, 1443 cm^−1^, 1346 cm^−1^, and 1257 cm^−1^ correspond to the O-H, C-H, C=H, C=C, N-H, C-O-C, and C-N functional groups, respectively. The presence of these functional groups confirms that both y-GQDs and c-GQDs possess a substantial amount of amino and hydroxyl groups on their surfaces. These amino and hydroxyl groups are highly reactive and can interact strongly with dye molecules through hydrogen bonds, charge interactions, or covalent bonding. These interactions facilitate the adsorption of dye molecules on the surface of GQDs, thereby enhancing the sensitivity and efficiency of dye detection. Figure 2c shows the Raman spectra of c-GQDs and y-GQDs. The D band (1305 cm^−1^) of c-GQDs corresponds to the disordered sp^3^ hybridized carbon structure, while the G band (1580 cm^−1^) corresponds to the ordered sp^2^ graphite carbon structure. Similarly, the D band (1360 cm^−1^) and G band (1585 cm^−1^) of y-GQDs were observed. By comparing the ID/IG ratio, it is evident that the ID/IG ratio of y-GQDs is lower than that of c-GQDs, further indicating that the graphitization degree of c-GQDs is lower than that of y-GQDs.

Nitrogen-doped GQDs (N-GQDs) serve as effective fluorescent probes for the highly sensitive detection of selected azo dyes. As shown in Table S1, the nitrogen content of c-GQDs is lower than that of y-GQDs, which is why y-GQDs were chosen as the GQDs for dye detection. Figure 2d–f present the time-resolved fluorescence decay curves of y-GQDs. It is observed that prior to the addition of MO, the fluorescence lifetime decay time is 3.946 ns, whereas after the addition of MO, the decay time decreases to 3.246 ns. This indicates that the fluorescence of y-GQDs can be efficiently quenched by MO.

### 3.3. The Optical Properties Characterization of GQDs

**Figure 3:**
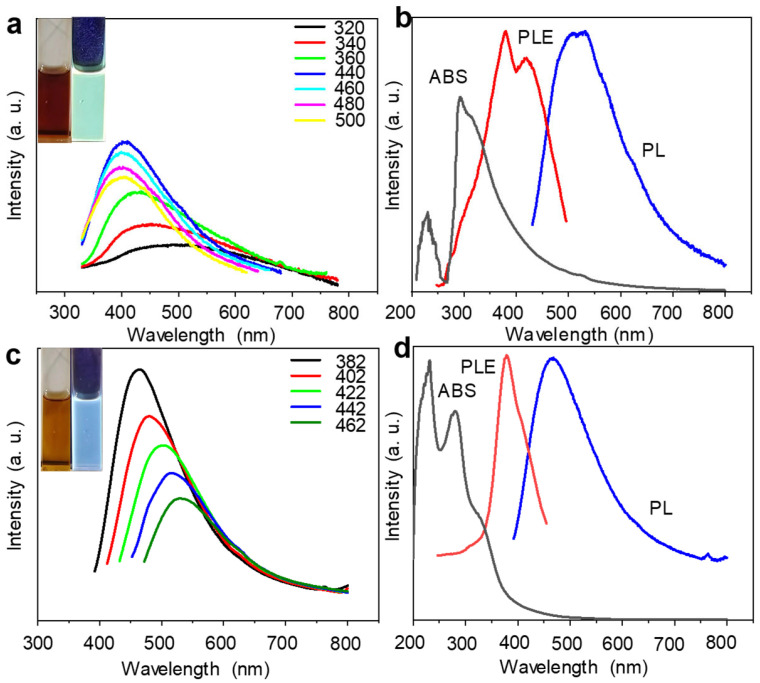
(**a**) Excitation wavelength dependence of y-GQDs; (**b**) photoluminescence (PL), photoluminescence excitation (PLE), and UV absorption spectra of y-GQDs; (**c**) excitation wavelength dependence of c-GQDs; (**d**) photoluminescence (PL), photoluminescence excitation (PLE), and UV absorption spectra of c-GQDs.

Figure 3d,b present the ultraviolet absorption and photoluminescence (PL) spectra of two types of GQDs, namely c-GQDs and y-GQDs. The maximum absorption peaks for c-GQDs and y-GQDs are observed at 280 nm and 300 nm, respectively, which can be attributed to the π–π* transitions of conjugated carbon–carbon double bonds. Additionally, as shown in Figure 3c,a, both c-GQDs and y-GQDs exhibit a brown color under natural light. Upon UV irradiation, c-GQDs emit cyan light, while y-GQDs emit pale yellow light, indicating the generation of fluorescence in both types of GQDs after the hydrothermal reaction. The polar solvent DMF facilitates the better dispersion of the GQDs and introduces more polar functional groups on their surface, thereby influencing their optical properties, such as absorption and emission wavelengths. In contrast, ethanol, being relatively non-polar, may induce different surface modifications and aggregation behaviors, leading to changes in optical characteristics and distinct color emissions. In summary, as shown in Figure 3b,d, PLE spectra are a complementary tool to PL spectra and are crucial for interpreting the material’s absorption properties. By carefully analyzing the trends and comparing PLE with absorption spectra, one can identify the origins of multiple peak features in PL spectra. This includes distinguishing between different types of electronic transitions, excitonic behaviors, and the roles of defect or trap states; the optimal excitation wavelength for c-GQDs is 380 nm, while for y-GQDs, it is 450 nm. As shown in Figure 3a,c, the fluorescence intensity peaks vary with changes in the excitation wavelength, suggesting that the PL spectra of c-GQDs and y-GQDs are independent of the excitation wavelength, owing to their highly ordered graphite structures.

**Figure 4:**
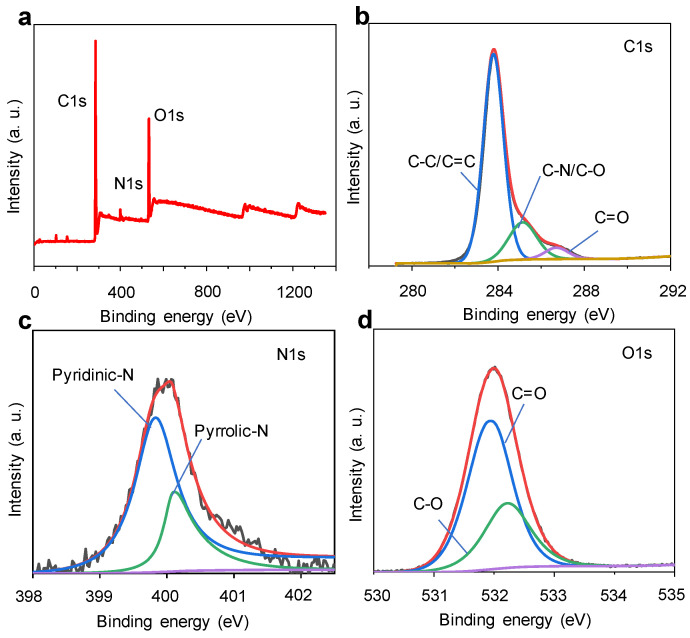
(**a**) Full XPS spectrum of y-GQDs; (**b**–**d**) high-resolution C 1s, N 1s, and O 1s spectra of y-GQDs.

The c-GQDs presented in Supplementary Materials Figure S1 follow the same principle. As shown in Figure 4, panel 4a displays the overall XPS spectrum of y-GQDs, confirming that the quantum dots are composed of carbon (C), nitrogen (N), and oxygen (O) elements, and were synthesized using orange peel as a precursor. Panel 4b shows the detailed C 1s spectrum, with peaks located at 283.5, 285.6, and 286.2 eV, which are attributed to C–C/C=C, C–N/C–O, and C=O bonds, respectively. Panel 4d presents the O 1s spectrum, which exhibits two peaks at 531.5 and 532.6 eV, indicating the presence of C–O and C=O bonds. Both FTIR and XPS analyses reveal the existence of hydrophilic functional groups, such as –OH, –COOH, and –NH2, on the surface of the GQDs, demonstrating their excellent water solubility. The fine N 1s spectra of the two quantum dots reveal that c-GQDs exhibit two peaks at 399.5 eV and 400.4 eV, while y-GQDs show peaks at 399.5 eV and 400 eV, which correspond to pyridinic nitrogen and pyrrolic nitrogen, respectively. Pyridinic nitrogen, with its higher electronegativity, tends to attract electrons, displaying an electron-withdrawing effect and acting as an electron-withdrawing group. In contrast, pyrrolic nitrogen tends to donate lone-pair electrons to the carbon atoms in the ring through resonance effects, thereby enhancing the electron-donating capacity of the molecule, making it an electron-donating group. As shown in Table S2, the pyridinic nitrogen and pyrrolic nitrogen ratio in c-GQDs is 65.07% and 34.93%, respectively, while in y-GQDs, the pyridinic nitrogen and pyrrolic nitrogen ratio is 76.57% and 23.43%. The pyridinic nitrogen-to-pyrrolic nitrogen ratio in c-GQDs is 1.86, while in y-GQDs, it is 3.26, indicating that y-GQDs possess a higher number of electron-withdrawing groups, which contributes to the observed redshift. As indicated in Table S1, the nitrogen content of c-GQDs is 2.82%, while that of y-GQDs is 3.35%, suggesting that GQDs prepared with DMF as a pure solvent can incorporate a higher nitrogen content.

### 3.4. The Application of GQDs in Dye Detection

**Figure 5:**
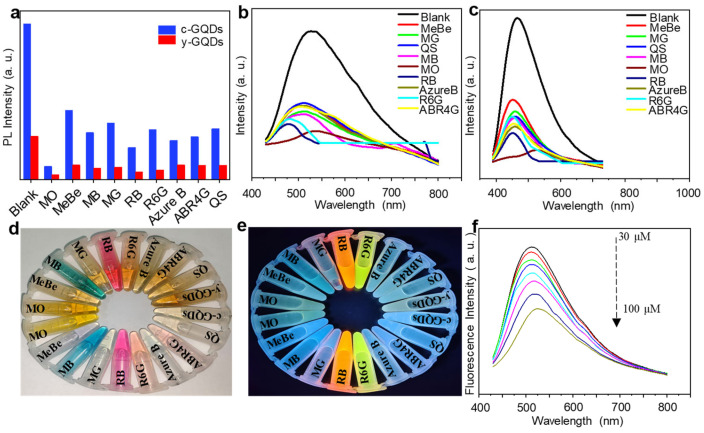
(**a**) Fluorescence intensity histograms of c-GQDs and y-GQDs in different dye solutions. (**b**) Fluorescence spectra of c-GQDs and (**c**) y-GQDs in various dye solutions. Images of y-GQDs and c-GQDs in different dye solutions under UV light (**e**) and natural light (**d**). (**f**) Variation in the luminescence intensity of y-GQDs at different methyl orange concentrations.

The dye selectivity of GQDs was subsequently evaluated. In this experiment, several common dye solutions were selected and prepared at a concentration of 0.1 mM for testing. c-GQDs and y-GQDs were then added to these solutions to assess fluorescence intensity and observe fluorescence quenching. As shown in Figure 5a, after the addition of c-GQDs and y-GQDs, c-GQDs exhibited noticeable fluorescence quenching only with methyl orange, whereas y-GQDs experienced fluorescence quenching with all tested dyes, with the quenching effect being most pronounced for methyl orange. In the presence of methyl orange, the fluorescence intensity of y-GQDs decreased to a very low level, while the fluorescence intensity of c-GQDs was much higher than that of y-GQDs. This demonstrates that y-GQDs exhibit significant fluorescence quenching in methyl orange solution and thus can be used for methyl orange detection. The fluorescence intensity data shown in Figure 5b (for c-GQDs with different dye solutions) and Figure 5c (for y-GQDs with different dye solutions) further support these findings. Figure 5e,d present the corresponding experimental results, where it is evident that c-GQDs undergo fluorescence quenching with methyl orange, while no significant quenching is observed with other dye solutions. In contrast, y-GQDs experience fluorescence quenching with all dye solutions, with the quenching effect being more pronounced, making y-GQDs suitable for methyl orange dye detection. The fluorescence intensity of y-GQDs and its linear correlation with the concentration of methyl orange were examined as the methyl orange concentration increased from 30 μM to 100 μM. As shown in Figure 5f, the fluorescence intensity of y-GQDs progressively decreased with the increasing concentration of methyl orange, ranging from 30 μM to 100 μM. Additionally, Figure S2 demonstrates a strong linear relationship between the concentration of methyl orange and the PL fluorescence intensity of y-GQDs, with a high correlation coefficient (R^2^ = 0.9954), indicating that y-GQDs exhibit excellent sensitivity for the detection of methyl orange. Table S3 provides a comparative analysis of various methods for detecting methyl orange, with graphene quantum dot-based detection emerging as the most preferred technique.

Supplementary Figure S3 presents the Zeta potential measurements of two types of quantum dots, both initially at a pH of 7. Upon the addition of acid and base, the Zeta potential was observed to change with increasing concentrations of H⁺ and OH⁻. At neutral pH, the Zeta potential of c-GQDs was measured at +7, while that of y-GQDs was +4, indicating that the Zeta potential of c-GQDs is higher than that of y-GQDs. This higher Zeta potential leads to better dispersion of c-GQDs in solution, reducing the likelihood of contact with the dye and thereby decreasing the potential for fluorescence quenching. In contrast, y-GQDs are more susceptible to fluorescence quenching by methyl orange, making them suitable for use in detecting this dye.

**Figure 6:**
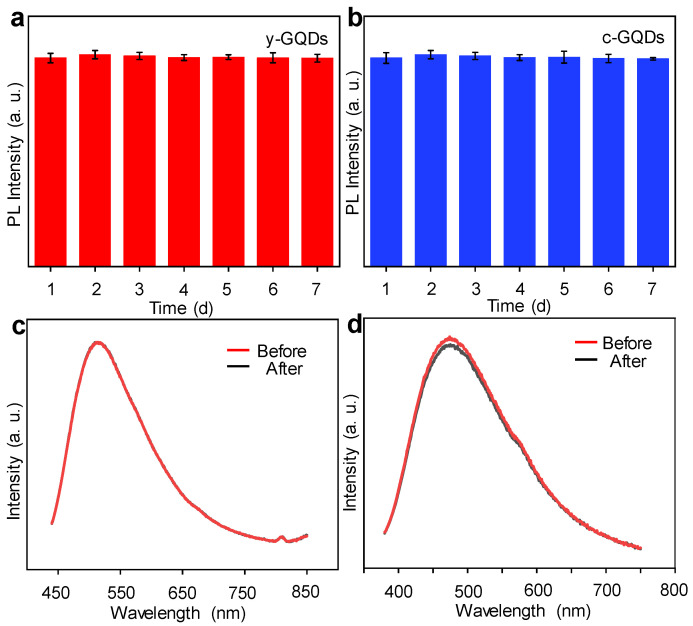
Changes in fluorescence intensity of (**a**) y-GQDs and (**b**) c-GQDs after seven days of placement. (**c**) Dispersion stability of y-GQDs; (**d**) dispersion stability of c-GQDs.

Next, we evaluated the stability of these two GQDs in terms of pH stability, dispersion stability, and time stability. Figure S4 presents the pH stability test for both quantum dots, starting with an initial pH of 7 and varying the pH by adding different concentrations of acids and bases. The fluorescence intensity was measured at each pH value. As shown in Figure S4, y-GQDs exhibit higher tolerance to acidic and basic conditions compared to c-GQDs. Ethanol molecules contain both a polar hydroxyl group (-OH) and a non-polar alkyl group (-CH₂CH₃). Although ethanol is a polar solvent, its polarity is weaker than that of DMF, making it less effective in dissolving polar molecules or charged particles. Ethanol’s hydrophilic nature also enables stronger hydrogen bonding interactions with acidic and basic components in aqueous solutions, potentially disrupting the pH balance and affecting the quantum dots’ stability. However, both types of GQDs show limited tolerance to alkaline conditions. The fluorescence intensity of y-GQDs remains relatively stable across a range of pH values, suggesting their acid resistance. c-GQDs also demonstrate some degree of acid resistance, but y-GQDs perform better, likely due to the influence of DMF as the solvent, which enhances the properties of y-GQDs. Figure 6a,b show the fluorescence intensity of the two quantum dots after being stored for seven days. The data reveal that the fluorescence intensity of both kinds of GQDs does not change significantly over time, indicating their good temporal stability. Figure 6c,d show the dispersion stability of the two GQDs. The fluorescence intensity of a 5 mL solution of each kind of GQD was measured before and after drying and redispersing in an equal volume of deionized water. The results from Figure 6d,c indicate that both c-GQDs and y-GQDs exhibit a minimal change in fluorescence intensity before and after redispersion, confirming their good dispersion stability.

## 4. Conclusions

In this study, common household waste, specifically orange peels, was selected as a precursor, and dimethylformamide (DMF) and ethanol were used as solvents to synthesize yellow y-GQDs and cyan c-GQDs via a bottom-up solvothermal method. The difference in color is attributed to the redshift caused by the varying ratio of pyridine nitrogen to pyrrole nitrogen. The morphology, structure, and properties of the resulting quantum dots were thoroughly characterized. The y-GQDs synthesized with DMF as the solvent exhibited an interlayer spacing of 3.12 Å, a diffraction angle around 26°, an average thickness of 3.51 nm, and a lattice spacing of 0.22 nm, demonstrating excellent performance. For application purposes, several common dye solutions were added to the quantum dots, and it was observed that methyl orange induced significant fluorescence quenching in both c-GQDs and y-GQDs. Upon the addition of methyl orange, the fluorescence intensity of y-GQDs decreased significantly, much more than that of c-GQDs, indicating that y-GQDs undergo substantial fluorescence quenching in the presence of methyl orange, making them suitable for detecting methyl orange dye. This experiment demonstrates that y-GQDs can serve as effective probes for methyl orange detection. Compared to other studies, this approach offers a low-cost and readily available precursor, and the use of pure solvents simplifies the solvothermal process, eliminating the need for complex hydrothermal adjustments and thus enabling the straightforward synthesis of yellow-light-emitting GQDs. The findings of this study highlight the potential of GQDs in chemical sensing applications, suggesting that they will become increasingly researched and utilized in the field of sensors in the future.

## Data Availability

Data will be made available upon request.

## Supplementary Materials

The following supporting information can be downloaded at https://www.mdpi.com/article/10.3390/nano15050376/s1: Figure S1: (a) XPS spectra of c-GQDs and (b–d) C, N, and O fine spectra of c-GQDs; Figure S2: regression equation; Figure S3: Zeta potentials of (a) y-GQDs and (b) c-GQDs; Figure S4: Fluorescence intensities at different pH (a) y-GQDs, (b) c-GQDs; Table S1: XPS measures the element ratios of c-GQDs and y-GQDs in the spectra; Table S2: The ratio of pyridinic-N to pyrrolic-N in the XPS N 1s high-resolution spectra of the two quantum dots; Table S3: Comparison of methods for the detection of methyl oranges.

## References

[1] Cai J., Han G., Ren J., Liu C., Wang J., Wang X. (2022). Single-layered graphene quantum dots with self-passivated layer from xylan for visual detection of trace chromium(Vl). Chem. Eng. J..

[2] Hu X.-H., Zhang R., Wu Z., Xiong S. (2022). Concentrated Solar Induced Graphene. ACS Omega.

[3] Ali O.M., Hasanin M.S., Suleiman W.B., Helal E.E.-H., Hashem A.H. (2022). Green biosynthesis of titanium dioxide quantum dots using watermelon peel waste: Antimicrobial, antioxidant, and anticancer activities. Biomass Convers. Biorefinery.

[4] Gong D., Guo J., Wang F., Zhang J., Song S., Feng B., Zhang X., Zhang W. (2023). Green construction of metal- and additive-free citrus peel-derived carbon dot/g-C3N4 photocatalysts for the high-performance photocatalytic decomposition of sunset yellow. Food Chem..

[5] Singh A.K., Sri S., Garimella L.B.V.S., Dhiman T.K., Sen S., Solanki P.R. (2022). Graphene Quantum Dot-Based Optical Sensing Platform for Aflatoxin B1 Detection via the Resonance Energy Transfer Phenomenon. ACS Appl. Bio Mater..

[6] Liu J., Li R., Yang B. (2020). Carbon Dots: A New Type of Carbon-Based Nanomaterial with Wide Applications. ACS Cent. Sci..

[7] Kumar J.V., Rhim J.-W. (2024). Fluorescent carbon quantum dots for food contaminants detection applications. J. Environ. Chem. Eng..

[8] Kolhe P., Roberts A., Gandhi S. (2023). Fabrication of an ultrasensitive electrochemical immunosensor coupled with biofunctionalized zero-dimensional graphene quantum dots for rapid detection of cephalexin. Food Chem..

[9] Gozali Balkanloo P., Mohammad Sharifi K., Poursattar Marjani A. (2023). Graphene quantum dots: Synthesis, characterization, and application in wastewater treatment: A review. Mater. Adv..

[10] Kadyan P., Malik R., Bhatia S., Al Harrasi A., Mohan S., Yadav M., Dalal S., Ramniwas S., Kumar Kataria S., Arasu T. (2023). Comprehensive Review on Synthesis, Applications, and Challenges of Graphene Quantum Dots (GQDs). J. Nanomater..

[11] Kundu A., Maity B., Basu S. (2023). Orange Pomace-Derived Fluorescent Carbon Quantum Dots: Detection of Dual Analytes in the Nanomolar Range. ACS Omega.

[12] Wu J., Chen T., Ge S., Fan W., Wang H., Zhang Z., Lichtfouse E., Van Tran T., Liew R.K., Rezakazemi M. (2023). Synthesis and applications of carbon quantum dots derived from biomass waste: A review. Environ. Chem. Lett..

[13] Fu S., Tang B., Wang Z., An G., Zhang M., Wang K., Liu W., Guo H., Zhang B., Wang L. (2024). Hydrophobic carbon quantum dots with Lewis-Basic nitrogen sites for electrocatalyst CO2 reduction to CH4. Chem. Eng. J..

[14] Pothipor C., Jakmunee J., Bamrungsap S., Ounnunkad K. (2021). An electrochemical biosensor for simultaneous detection of breast cancer clinically related microRNAs based on a gold nanoparticles/graphene quantum dots/graphene oxide film. Anal..

[15] Liu X., Deng J., Li J., Dong J., Liu H., Zhao J., Luo X., Huo D., Hou C. (2023). B-doped graphene quantum dots array as fluorescent sensor platforms for plasticizers detection. Sens. Actuators B Chem..

[16] Tang S., Chen D., Yang Y., Wang C., Li X., Wang Y., Gu C., Cao Z. (2022). Mechanisms behind multicolor tunable Near-Infrared triple emission in graphene quantum dots and ratio fluorescent probe for water detection. J. Colloid Interface Sci..

[17] Nair R.V., Chandran P.R., Mohamed A.P., Pillai S. (2021). Sulphur-doped graphene quantum dot based fluorescent turn-on aptasensor for selective and ultrasensitive detection of omethoate. Anal. Chim. Acta.

[18] Xie L., Liang C., Wu Y., Wang K., Hou W., Guo H., Wang Z., Lam Y.M., Liu Z., Wang L. (2024). Isomerization Engineering of Oxygen-Enriched Carbon Quantum Dots for Efficient Electrochemical Hydrogen Peroxide Production. Small.

[19] Wang C., Chen D., Yang Y., Tang S., Li X., Xie F., Wang G., Guo Q. (2021). Synthesis of multi-color fluorine and nitrogen co-doped graphene quantum dots for use in tetracycline detection, colorful solid fluorescent ink, and film. J. Colloid Interface Sci..

[20] Hu B., Huang K., Tang B., Lei Z., Wang Z., Guo H., Lian C., Liu Z., Wang L. (2023). Graphene Quantum Dot-Mediated Atom-Layer Semiconductor Electrocatalyst for Hydrogen Evolution. Nano-Micro Lett..

[21] Liu H., Deng Z., Zhang Z., Lin W., Zhang M., Wang H. (2024). Graphene quantum dots as metal-free nanozymes for chemodynamic therapy of cancer. Matter.

[22] Anusuya T., Kumar V., Kumar V. (2021). Hydrophilic graphene quantum dots as turn-off fluorescent nanoprobes for toxic heavy metal ions detection in aqueous media. Chemosphere.

[23] Duprez H., Cances S., Omahen A., Masseroni M., Ruckriegel M.J., Adam C., Tong C., Garreis R., Gerber J.D., Huang W. (2024). Spin-valley locked excited states spectroscopy in a one-particle bilayer graphene quantum dot. Nat. Commun..

[24] Ni Q., Zhang S., Wang K., Guo H., Zhang J., Wu M., Wang L. (2024). Carbon quantum dot-mediated binary metal–organic framework nanosheets for efficient oxygen evolution at ampere-level current densities in proton exchange membrane electrolyzers. J. Mater. Chem. A.

[25] Li G., Liu Z., Gao W., Tang B. (2023). Recent advancement in graphene quantum dots based fluorescent sensor: Design, construction and bio-medical applications. Coord. Chem. Rev..

[26] Zhu L., Li D., Lu H., Zhang S., Gao H. (2022). Lignin-based fluorescence-switchable graphene quantum dots for Fe3+ and ascorbic acid detection. Int. J. Biol. Macromol..

[27] Tam T.V., Hur S.H., Chung J.S., Choi W.M. (2021). Novel paper- and fiber optic-based fluorescent sensor for glucose detection using aniline-functionalized graphene quantum dots. Sens. Actuators B Chem..

[28] Kang S., Han H., Lee K., Kim K.M. (2022). Ultrasensitive Detection of Fe3+ Ions Using Functionalized Graphene Quantum Dots Fabricated by a One-Step Pulsed Laser Ablation Process. ACS Omega.

[29] Revesz I.A., Hickey S.M., Sweetman M.J. (2022). Metal ion sensing with graphene quantum dots: Detection of harmful contaminants and biorelevant species. J. Mater. Chem. B.

[30] Wang R., Jiao L., Zhou X., Guo Z., Bian H., Dai H. (2021). Highly fluorescent graphene quantum dots from biorefinery waste for tri-channel sensitive detection of Fe3+ ions. J. Hazard. Mater..

[31] Huynh T.V., Anh N.T.N., Darmanto W., Doong R.-A. (2021). Erbium-doped graphene quantum dots with up- and down-conversion luminescence for effective detection of ferric ions in water and human serum. Sens. Actuators B Chem..

[32] Reagen S., Wu Y., Liu X., Shahni R., Bogenschuetz J., Wu X., Chu Q.R., Oncel N., Zhang J., Hou X. (2021). Synthesis of Highly Near-Infrared Fluorescent Graphene Quantum Dots Using Biomass-Derived Materials for In Vitro Cell Imaging and Metal Ion Detection. ACS Appl. Mater. Interfaces.

[33] He W., Lim S.F., Baini R., Qu Y., Li X. (2024). Visual Determination of Copper (II) Using a Biomass Sourced Carbon Quantum Dot (CQD) Ratiometric Fluorescent Probe. Anal. Lett..

[34] Mousavi S.M., Hashemi S.A., Yari Kalashgrani M., Kurniawan D., Gholami A., Rahmanian V., Omidifar N., Chiang W.-H. (2022). Recent Advances in Inflammatory Diagnosis with Graphene Quantum Dots Enhanced SERS Detection. Biosensors.

[35] Michenzi C., Proietti A., Rossi M., Espro C., Bressi V., Vetica F., Simonis B., Chiarotto I. (2024). Carbon nanodots from orange peel waste as fluorescent probes for detecting nitrobenzene. RSC Sustain..

[36] Karthikeyan M., Hemalatha M., Venkatasubbu D.G., Abimanyu S., Aravind M., Rathinsabapathi P. (2025). Label-free detection of ampicillin in milk and water using fluorescence carbon dots synthesised from Citrus Limetta peel. Diam. Relat. Mater..

[37] Sun Y., Ma S., Wang H., Wang H., Gao M., Wang X. (2023). Construction of an “ON–OFF” fluoroprobe using ionic liquids-modified orange peel-based carbon quantum dots for selective/sensitive permanganate assay in waters and the underlying quenching mechanisms. Anal. Bioanal. Chem..

[38] Gholipour A., Rahmani S. (2023). The Green Synthesis of Carbon Quantum Dots through One-step Hydrothermal Approach by Orange Juice for Rapid, and Accurate Detection of Dopamine. J. Fluoresc..

[39] Surendran P., Lakshmanan A., Priya S.S., Balakrishnan K., Rameshkumar P., Kannan K., Mahalakshmi K., Gayathri V., Vinitha G. (2024). Synthesis of fluorescent carbon quantum dots from Manihot esculenta waste peels for nonlinear optical and biological applications. Chem. Phys. Impact.

[40] Li M., Jiang T., Wang X., Chen H., Li S., Wei F., Ren Z., Yan S., Guo X., Tu H. (2020). Preparation of highly oriented single crystal arrays of C8-BTBT by epitaxial growth on oriented isotactic polypropylene. J. Mater. Chem. C.

