# Supplementary material for: Fluorescence Quenching of Graphene Quantum Dots from Orange Peel for Methyl Orange Detection
Source: Nanomaterials (Basel). 2025 Feb 28;15(5):376. doi: 10.3390/nano15050376 (PMC11901586; doi:10.3390/nano15050376)
Supplement: Supplementary file 1 [file nanomaterials-15-00376-s001.zip › nanomaterials-3425835-supplementary.pdf]

# Supporting Information

# Fluorescence Quenching of Graphene Quantum Dots from Orange Peel for Methyl Orange Detection

**Weitao Li [1,2], Yang Liu [1,2], Xinglong Pang [3,4], Yuanhao Huang [1,2], Zeyun Dong [1,2], Qian Niu [1,2], Yuping Xiong [1,2], Shang Li [1,2], Shuai Li [1,2], Lei Wang [1,*], Huazhang Guo [3,*], Saisai Cui [1,2], Shenpeng Hu [1,2], Yuenan Li [1,2], Tiantian Cha [1,2] and Liang Wang [3]**

[1] Textile and Garment Industry of Research Institute, Zhongyuan University of Technology, Zhengzhou 450007, China
[2] Zhengzhou Key Laboratory of Smart Fabrics & Flexible Electronics Technology, Zhongyuan University of Technology, Zhengzhou 451191, China
[3] Institute of Nanochemistry and Nanobiology, School of Environmental and Chemical Engineering, Shanghai University, Shanghai 200444, China
[4] Department of Environment, Yangtze Delta Region Institute of Tsinghua University, Jiaxing 314006, China
* Correspondence: wanglei@zut.edu.cn (L.W.); guohuazhang@shu.edu.cn (H.G.)

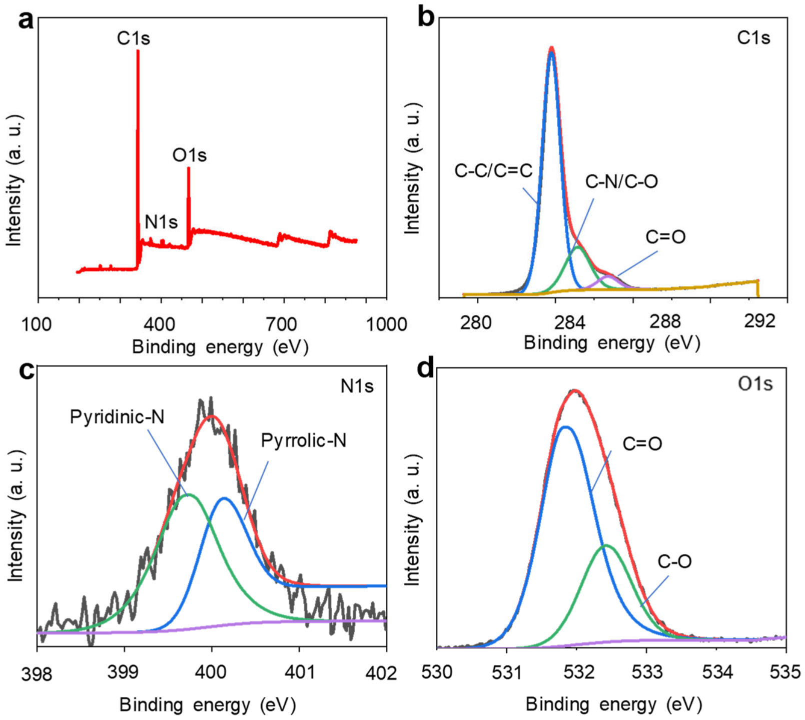


**Figure S1** (a) XPS spectra of c-GQDs and (b-d) C, N, and O fine spectra of c-GQDs.

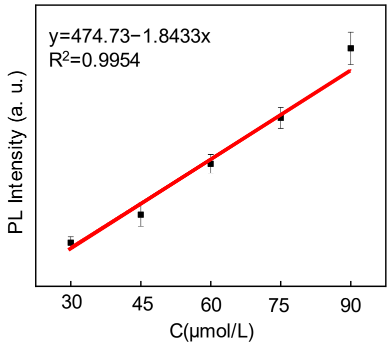


**Figure S2** regression equation.

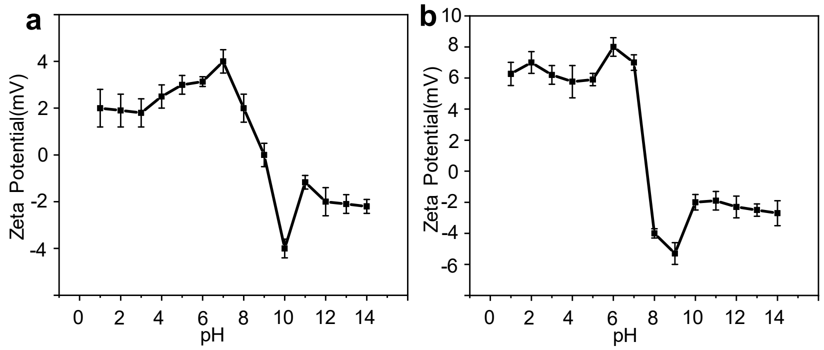


**Figure S3** Zeta potentials of (a) y-GQDs and (b) c-GQDs.

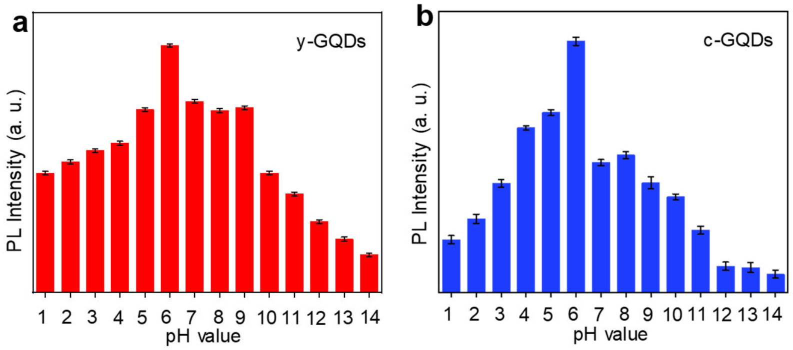


**Figure S4** Fluorescence intensities at different pH (a) y-GQDs, (b) c-GQDs.

**Table S1.** XPS measures the element ratios of c-GQDs and y-GQDs in the spectra.

| **Element** | **C** | **N** | **O** |
|---|---|---|---|
| c-GQDs | 79.35 | 2.81 | 17.84 |
| y-GQDs | 76.79 | 3.36 | 19.87 |

**Table S2.** The ratio of pyridinic-N to pyrrolic-N in the XPS N 1s high-resolution spectra of the two quantum dots.

| Element | Pyridinic-N | Pyrrolic-N |
|---|---|---|
| c-GQDs | 65.07 | 34.93 |
| y-GQDs | 76.57 | 23.43 |

**Table S3.** Comparison of methods for the detection of methyl oranges

| Method | Advantage | Detection range | Limitations |
|---|---|---|---|
| UV-Vis spectrophotometry | Easy to operate | 1μM-100μM | The equipment is expensive and has low sensitivity to the sample |
| High Performance Liquid Chromatography | High precision sensitivity | 1μM-100μM | The equipment is expensive and the operation is complicated |
| Electrochemical sensor method | High sensitivity and fast response | 10μM-50μM | High requirements for equipment parameters |
| Fluorescence method | High sensitivity | 1μM-10μM | Prone to interference |
| Molecularly imprinted polymer sensor method | High selectivity | 1μM-10μM | Low detection limit, complex preparation process, high cost |
| Graphene quantum dot detection | Adjustable and highly sensitive | 1μM-50μM | No need for complex equipment, low cost, environmental protection |
